# Dual-Task Interference Effects on Lower-Extremity Muscle Activities during Gait Initiation and Steady-State Gait among Healthy Young Individuals, Measured Using Wireless Electromyography Sensors

**DOI:** 10.3390/s23218842

**Published:** 2023-10-31

**Authors:** Ke’Vaughn Tarrel Waldon, Angeloh Stout, Kaitlin Manning, Leslie Gray, David George Wilson, Gu Eon Kang

**Affiliations:** 1 Department of Bioengineering, University of Texas at Dallas, Richardson, TX 75080, USAangeloh.stout@utdallas.edu (A.S.);; 2 Department of Prosthetics-Orthotics, University of Texas Southwestern Medical Center, Dallas, TX 75390, USA; 3 Department of Plastic Surgery, University of Texas Southwestern Medical Center, Dallas, TX 75390, USA

**Keywords:** gait initiation, dual task, muscle activities, wireless electromyography

## Abstract

To maintain a healthy lifestyle, adults rely on their ability to walk while simultaneously managing multiple tasks that challenge their coordination. This study investigates the impact of cognitive dual tasks on lower-limb muscle activities in 21 healthy young adults during both gait initiation and steady-state gait. We utilized wireless electromyography sensors to measure muscle activities, along with a 3D motion capture system and force plates to detect the phases of gait initiation and steady-state gait. The participants were asked to walk at their self-selected pace, and we compared single-task and dual-task conditions. We analyzed mean muscle activation and coactivation in the biceps femoris, vastus lateralis, gastrocnemius, and tibialis anterior muscles. The findings revealed that, during gait initiation with the dual-task condition, there was a decrease in mean muscle activation and an increase in mean muscle coactivation between the swing and stance limbs compared with the single-task condition. In steady-state gait, there was also a decrease in mean muscle activation in the dual-task condition compared with the single-task condition. When participants performed dual-task activities during gait initiation, early indicators of reduced balance capability were observed. Additionally, during dual-task steady-state gait, the knee stabilizer muscles exhibited signs of altered activation, contributing to balance instability.

## 1. Introduction

In daily life, individuals often encounter situations where they need to simultaneously perform additional tasks during walking, known as dual-task (DT) walking. These additional tasks can include cognitive activities, such as problem-solving. Walking itself, also known as single-task (ST) walking, requires the integration and coordination of various motor and cognitive processes, including attention to the environment and neuro-biomechanical control of body segments during locomotion [1,2,3,4]. However, when additional cognitive activities are introduced during walking, they interfere with the execution of fundamental balance control, resulting in what is known as DT interference [5,6,7]. This interference presents further challenges to an individual’s walking performance and may increase the risk of injuries, such as falls [8]. For this reason, researchers have focused on investigating the extent to which balance control is disrupted during DT walking compared with ST walking [9,10,11].

Numerous studies have examined spatiotemporal gait parameters during DT and ST walking, consistently revealing slower gait speed, shorter stride length, and greater gait variability (i.e., increased stride-to-stride fluctuations in spatiotemporal gait parameters) during DT walking. These findings remain consistent across various age groups and clinical conditions [12,13,14]. While fewer studies have investigated lower-extremity joint kinematics during DT walking, recent research by Piche et al. reported differences in angular velocities at the hip and knee between DT and ST walking in older adults [15]. Additionally, Ko et al. found that older adults unable to perform DT walking exhibited reduced ranges of motion in the knee and ankle compared with their counterparts capable of performing DTs [16]. These findings suggest disruptions in lower-extremity movement during DT walking, potentially contributing to differences in spatiotemporal gait parameters. Although results from these reports may be slightly mixed, they provide implications that there may be some disruptions in lower-extremity movement during DT walking compared with ST walking, which may have resulted in differences in spatiotemporal gait parameters. This further suggests that lower-extremity muscle activity may have contributed more fundamental neuromuscular causes to disrupt the lower-extremity movement [17], and yet studies investigating lower-extremity muscle activity during DT walking compared with ST walking are scarce, with only a recent study existing [18].

Furthermore, the effects of DT interference on neuromuscular control may have a more pronounced impact on locomotion tasks requiring substantial balance control, such as gait initiation (GI), compared with rhythmic and repetitive phases like steady-state (SS) gait [19,20,21,22]. However, to the best of our knowledge, no study has yet compared lower-extremity muscle activity during DT GI and ST GI. Therefore, this study aims to examine the effects of DT interference on lower-extremity muscle activity during both GI and SS gait in healthy young adults. We hypothesize that healthy young adults will have decreased lower-extremity muscle activity in both GI and SS gait during DT walking compared with ST walking.

## 2. Materials and Methods

### 2.1. Participants

All the experimental procedures were carried out at the Neuromuscular and Musculoskeletal Biomechanics Laboratory at the University of Texas at Dallas (UTD). Twenty-one healthy young adult volunteers from the UTD community participated in this study after providing signed informed consent, which was approved by the UTD Institutional Review Board. Inclusion criteria for participants included English proficiency, the ability to walk without any walking aids, good general health, and a body mass index (BMI) ranging from 18.5 to 29.9 kg/m^2^. The inclusion of English proficiency and BMI as criteria is based on the potential influence of English proficiency on performance in DT conditions and the impact of BMI on an individual’s gait pattern [23,24,25,26]. Exclusion criteria encompassed a history of musculoskeletal, neurological, or cardiovascular disease.

### 2.2. Experimental Protocol

After obtaining consent, we collected the participants’ demographic information. We performed Montreal Cognitive Assessment (MOCA) to evaluate participants’ cognitive status [27]. We asked the participants to change into tight-fitting clothing (short pants and short sleeves). We then attached 16-channel Delsys Trigno, wireless surface electromyography (EMG) sensors (Delsys Inc., Natick, MA, USA) bilaterally to the lower limbs. We followed the non-invasive assessment of muscles (SENIAM) guidelines for EMG attachment [28]. Briefly, the participant’s skin was cleaned using isopropyl alcohol to limit impedance. Once applied, the skin was allowed time to dry. Following skin preparation, the EMG sensors were cleaned with isopropyl alcohol and allowed to dry before placement. The associated thigh-segment lower-limb muscles (hamstring and quadricep muscles) were the vastus lateralis (VL) and biceps femoris (BF). The associated shank segment (lower knee and calf muscles) included the tibialis anterior (TA) and gastrocnemius.

**Figure 1:**
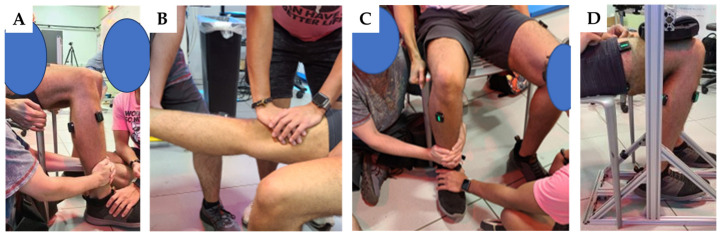
MVC trials. (**A**) In the MVC trial for the VL, participants were seated with all lower limb joints at 90 degrees. They were then instructed to extend the knee with maximum force while the assessor secured the shank. (**B**) In the MVC trial for the BF, participants were seated with the knee fully extended and then asked to flex the knee as forcefully as possible while the assessor held the shank and anchored a hand on the lower femur. (**C**) The MVC trial for the TA involved the participants being seated with all lower limb joints at 90 degrees. They were then instructed to dorsiflex the ankle with maximum effort while the assessor held down the toes on the foot. (**D**) For the MVC trial targeting the gastrocnemius, participants were seated with all lower limb joints at 90 degrees. They were asked to plantarflex the ankle with maximum force while the assessor held down the distal thigh.

Once all EMG sensors were attached, data from the maximum voluntary contraction (MVC) was collected by two consecutive trials for each muscle as a reference value to normalize EMG data from gait trials (Figure 1). For the MVC trials, participants were asked to sit at a 90-degree angle between the seat and lower limb joints for each muscle configuration [29]. During each trial, the participants were provided the following queues: to move the limb in the contracting direction (build up) from 0 to 3 s, exert as much force as possible (max) from 4 to 8 s, and stop the movement (relax) from 8 to 10 s. All EMG data (MVC trials and gait trials) were collected at a sampling rate of 2000 Hz.

**Figure 2:**
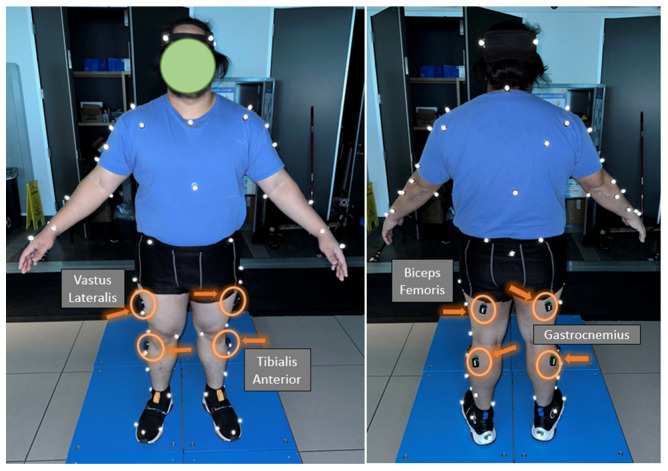
Placement of EMG sensors and reflective markers.

In addition to the EMG sensors, we used a 10-camera 3D motion capture system (Vicon Motion Systems Ltd., Oxford, UK) with a sampling rate of 100 Hz to identify GI and SS. For the motion capture, we placed 74 reflective markers on each participant’s full body: the clavicular notch (jugular notch), xiphisternum, along the spine C7 vertebrae, along the spine T10 vertebrae, apex of acromioclavicular joint bilaterally (including anterior and posterior), anterior superior iliac spine bilaterally, posterior superior iliac spine bilaterally, medial and lateral condyle of the femur, medial and lateral malleolus, calcaneus bilaterally, proximally to the medial and lateral epicondyle, center of the first and fifth metatarsals, and apex of proximal phalanx 1 (Figure 2). In addition, there were clustered markers placed on the upper arm, forearm, thigh, and tibia to track segments. The medial markers were removed after the static and bodyweight trials [30].

A reference static trial was conducted prior. In the reference trial, the participant was instructed to stand in anatomical pose (Figure 2). Once completed, we measured body weight using a single force plate (Kistler Instruments Ltd., Farnborough, UK). Then, the participant was allowed up to 5 min to understand the procedure for the tasks they would perform. During this time, the participants were able to become acquainted with the laboratory situation.

Subsequently, the gait trials for both the ST and DT conditions were conducted consecutively. Gait trials took place on a 10 m level ground walkway equipped with two force plates at the beginning and an additional two in the middle. For the ST condition, participants were instructed to stand naturally at the starting area of the walkway, with each foot positioned on one of the force plates. They were then asked to walk across the walkway at their self-selected speed, without any additional tasks (ST gait). In the DT condition, participants also began by standing naturally at the same starting point as in the ST condition. They were then instructed to verbally subtract numbers in a serial fashion, starting from a random number (e.g., subtracting 3 from 100) aloud. Participants commenced walking after subtracting one or two numbers and continued the subtraction task audibly throughout the gait trial (DT gait). The numerical subtraction task is a well-established method for investigating the effects of cognitive dual-tasking during walking and has been widely utilized in numerous previous studies [31,32,33,34]. The gait trials for each condition were repeated three times.

### 2.3. Data Analysis

We used Visual3D (C-motion, Germantown, MD, USA) to process the data from markers and force plates. These data were used to identify GI and SS gait. In brief, SS gait was estimated using markers placed at the foot, toe, heel, and sacrum following a previously published protocol [35].

For GI analysis, three parameters were considered. The first was onset, the duration of time until the vertical ground reaction force deviated above the mean of three standard deviations. This marked the initiation of gait movement. The second was weight transition, normally referred to as weight acceptance [35]. This represents the time taken from the initial contact of the stepping limb with the force plate until the vertical ground reaction force is fully shifted to that plate. This is the instance when the body transfers its weight from the initial stance limb to the stepping limb. The third was offset, the duration of time during which the stepping limb is swinging and the standing limb is reaching the instance of toe-off, showing significant reductions in force plate displacement, while preparing to take the next consecutive step. This concludes the process of GI. These data were obtained from motion capture and force plates following methods described previously to ensure the consistency and comparability of data analysis [35].

**Figure 3:**
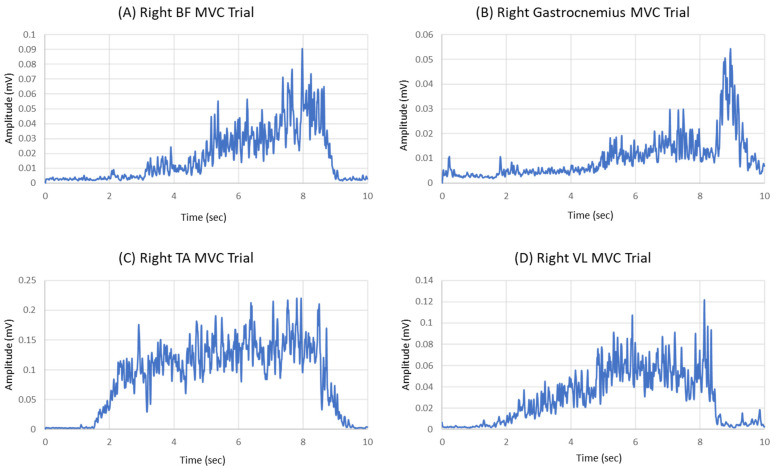
Example EMG data for MVC trials of biceps femoris (**A**), gastrocnemius (**B**), tibialis anterior (**C**), and vastus lateralis (**D**).

In SS gait, we analyzed the mean muscle activation by taking EMG signal data from the gait cycles in the motion trials. These gait cycles were created by Visual3D pipelines that calculated the foot strikes to toe-offs for each limb. In GI, we calculated the mean muscle activation and coactivation from EMG signal data from the onset to the offset phases of the motion trial. EMG data from GI and SS gait cycles were filtered in Visual3D. A band-pass filter (10–350 Hz) was applied to the EMG signals collected from the MVC and motion trials [36]. Then, a fourth-order filter with a cut-off frequency of 10 Hz was applied. The EMG signals were then full-wave-rectified. A linear envelope was applied by a 1 ms moving average window. EMG data were smoothed by normalizing the time to 1000 data point samples. The EMG data were processed in MATLAB R2022a (The MathWorks Inc., Natick, MA, USA). The peak EMG signal value was extracted from the MVC trials. From the MVC trials, the highest peak EMG signal value was extracted. To consider the MVC trial for later reference in data analysis, a peak value was taken within a 10 s time frame (Figure 3). The peak EMG mean value was taken from three motion trials from the muscles investigated in the thigh and shank.

The calculation of coactivation in gait initiation (GI) was performed using Equation (1), which is based on the activity of two specific muscles [37,38]. For example, the muscle activity in the BF and VL was calculated as follows (Equation (1)) [39,40]:

(1) $$Coactivation\;(\%)=\frac{LowEMGTrial}{HighEMGTrial}\times \left(LowEMGTrial+HighEMGTrial\right)\times 100$$

To calculate the percentage of coactivation, a custom MATLAB code was utilized, which divided the lower muscle activation by the higher muscle activation and then multiplied the result by the combined high and low activities, all multiplied by 100 [39].

### 2.4. Statistical Analysis

All participant data were statistically processed using SPSS software version 29.0 IBM. A paired *t*-test was used for comparing the muscle activation and coactivation between single and DT conditions. The means and standard deviation values were summarized and included in subject demographics. A Shapiro–Wilk test was performed to determine the normality of data samples. For non-normal data, a transformation was conducted through a logarithmic calculation. Following this, a paired *t*-test was conducted to obtain the two-tailed value. The Kruskal–Wallis test was performed for non-normal data. The sample significance was observed between the groups (*p* < 0.05). We calculated the group effect size with Cohen’s d (d). The group effect size represents d < 0.2 as a small effect, 0.2 ≤ d < 0.5 as a medium effect, and 0.5 ≤ d < 0.8 as a large effect [41].

## 3. Results

### 3.1. Participants

An analysis of 21 healthy young adults was conducted. Fourteen males and seven females participated in this study (Table 1). The subject demographics include the sample size (n), age (yrs), height (meters), weight (kg), BMI (kg/m^2^), MOCA (30 total points), and IPAQ (time expended partaking in different activities).

### 3.2. Gait Initiation

**Figure 4:**
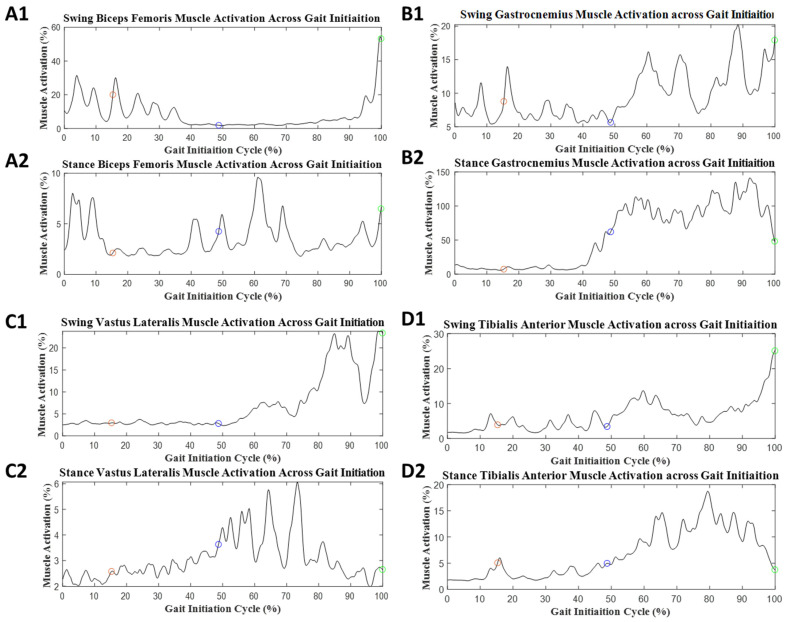
Average muscle activation during GI with the ST condition: (**A1**) biceps femoris muscle activity for swing limb, (**A2**) biceps femoris muscle activity for stance limb, (**B1**) gastrocnemius muscle activity for swing limb, (**B2**) gastrocnemius muscle activity for stance limb, (**C1**) vastus lateralis muscle activity for swing limb, (**C2**) vastus lateralis muscle activity for stance limb, (**D1**) tibialis anterior muscle activity for swing limb, and (**D2**) tibialis anterior muscle activity for stance limb. The red circle represents the end of the onset phase of GI. The blue circle represents the end of the weight transition phase of GI. The green circle represents the end of the offset phase of GI.

**Figure 5:**
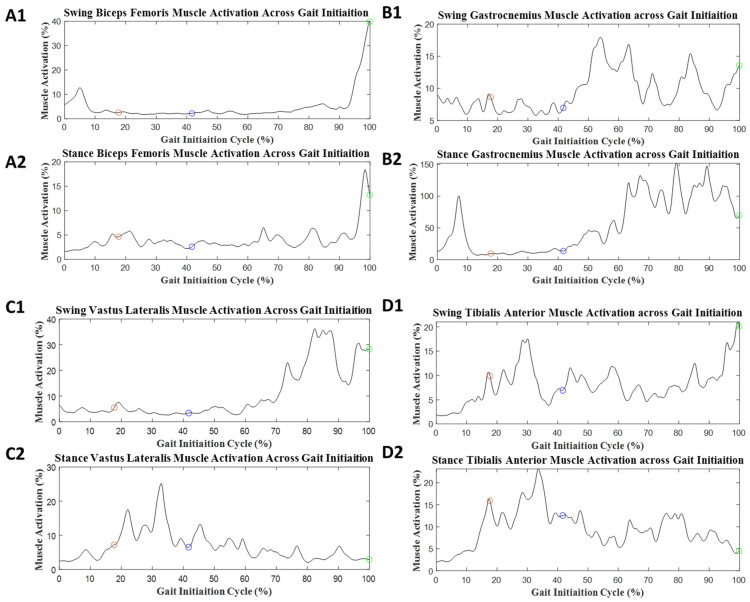
Average muscle activation during GI with the DT condition: (**A1**) biceps femoris muscle activity for swing limb, (**A2**) biceps femoris muscle activity for stance limb, (**B1**) gastrocnemius muscle activity for swing limb, (**B2**) gastrocnemius muscle activity for stance limb, (**C1**) vastus lateralis muscle activity for swing limb, (**C2**) vastus lateralis muscle activity for stance limb, (**D1**) tibialis anterior muscle activity for swing limb, and (**D2**) tibialis anterior muscle activity for stance limb. The red circle represents the end of the onset phase of GI. The blue circle represents the end of the weight transition phase of GI. The green circle represents the end of the offset phase of GI.

For GI, EMG data from 15 participants were analyzed due to protocol violation and technical issues. Examples of muscle activation during GI in both the ST and DT conditions are presented in Figure 4 and Figure 5, respectively, and the values for the mean muscle activity are provided in Table 2. The significant effect values were found in GI-phase events in mean muscle activation. The onset phase had a medium group effect, Stance BF (*p* = 0.071, d = 0.50). The weight transition phase had a medium group effect, Stance gastrocnemius (*p* = 0.088, d = 0.49). The offset phase significant values were Stance BF (*p* = 0.038, d = 0.59) and Stance TA (*p* < 0.001, d = 1.1). The full GI phase had significant values, Stance TA (*p* = 0.003, d = 0.91).

**Figure 6:**
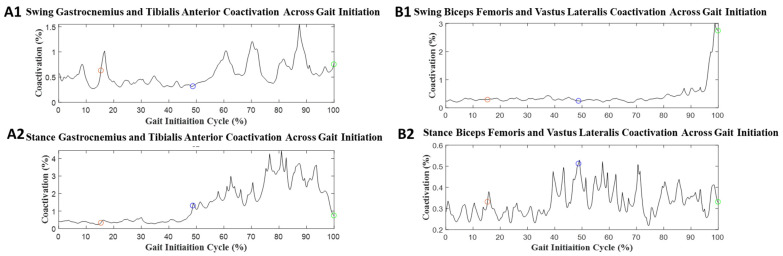
Mean muscle coactivation during ST GI: (**A1**,**A2**) tibialis anterior and gastrocnemius for swing and stance limbs, and (**B1**,**B2**) biceps femoris and vastus lateralis for swing and stance limbs. The black line represents the mean muscle coactivation as a normalized percentage for GI. The red circle represents the end of the onset phase of GI. The blue circle represents the end of the weight transition phase of GI. The green circle represents the end of the offset phase of GI.

**Figure 7:**
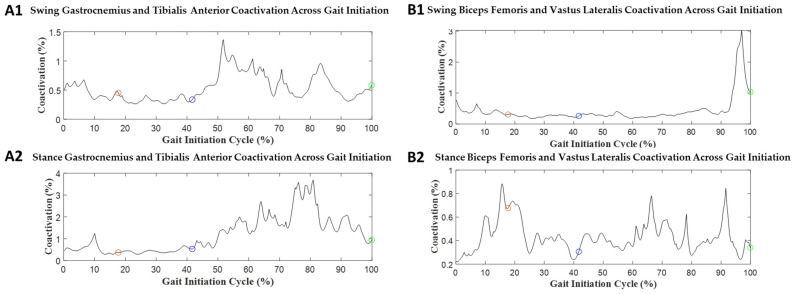
Mean muscle coactivation during DT GI: (**A1**,**A2**) tibialis anterior and gastrocnemius for swing and stance limbs, and (**B1**,**B2**) biceps femoris and vastus lateralis for swing and stance limbs. The black line represents the mean muscle coactivation as a normalized percentage for GI. The red circle represents the end of the onset phase of GI. The blue circle represents the end of the weight transition phase of GI. The green circle represents the end of the offset phase of GI.

Examples of muscle coactivation during GI in both the ST and DT conditions are presented in Figure 6 and Figure 7, respectively, and values for the mean muscle coactivation are provided in Table 3. Significant effect values were found in GI phase events in mean muscle coactivation. The onset phase had no significant differences. The weight transition phases’ significant values were: swing BF/VL (*p* = 0.14, d = 0.73), stance BF/VL (*p* = 0.004, d = 0.88), and stance gastrocnemius/TA (*p* = 0.043, d = 0.60). The offset phases’ significant values were: stance gastrocnemius/TA (*p* < 0.001, d = 1.1). The full GI had significant values in stance gastrocnemius/TA (*p* = 0.006, d = 0.84).

### 3.3. Steady-State Gait

**Figure 8:**
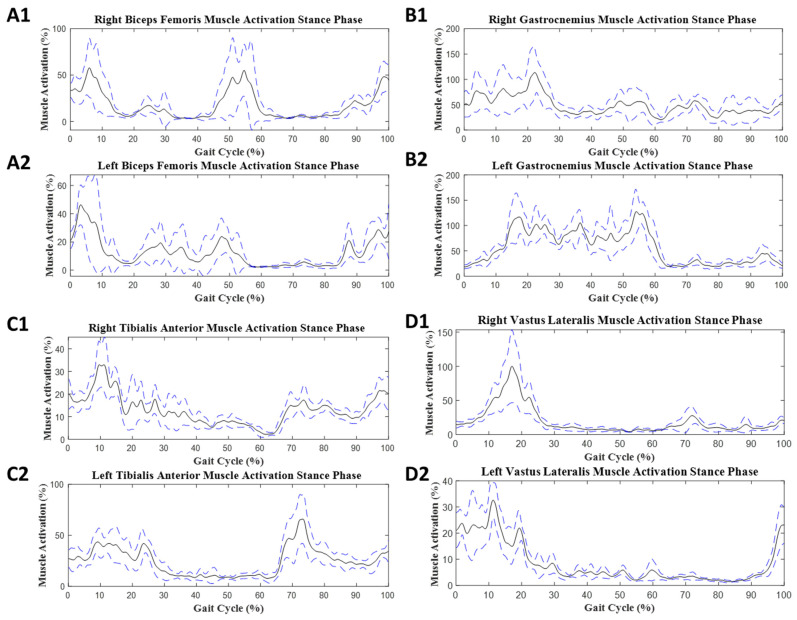
Average muscle activation during SS with the ST condition: (**A1**,**A2**) biceps femoris muscle activity, (**B1**,**B2**) gastrocnemius muscle activity, (**C1**,**C2**) tibialis anterior muscle activity, and (**D1**,**D2**) vastus lateralis muscle activity. The black solid line represents the mean muscle activation as a normalized percentage for the ST stance phase. The blue dashed line represents the high and low limits for the standard deviation.

**Figure 9:**
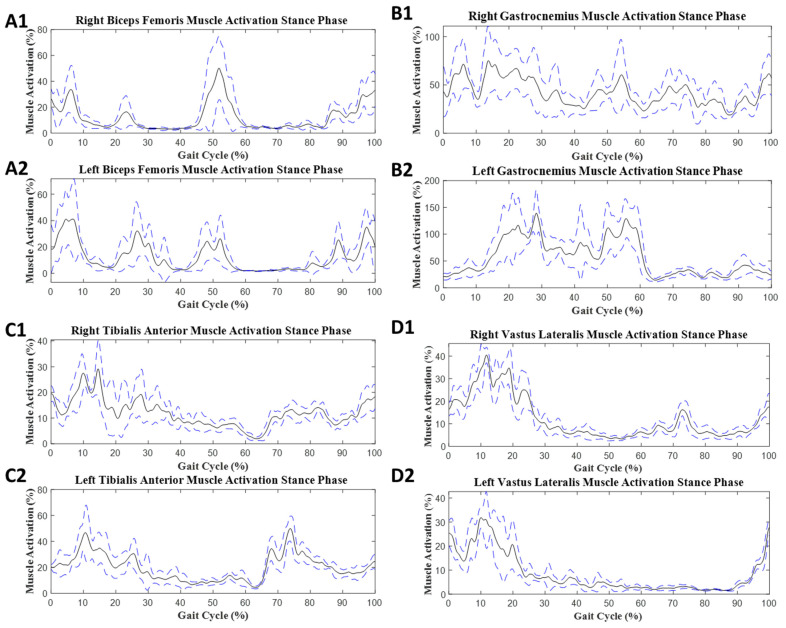
Average muscle activation during SS with the DT condition: (**A1**,**A2**) biceps femoris muscle activity, (**B1**,**B2**) gastrocnemius muscle activity, (**C1**,**C2**) tibialis anterior muscle activity, and (**D1**,**D2**) vastus lateralis muscle activity. The black solid line represents the mean muscle activation as a normalized percentage for the ST stance phase. The blue dashed line represents the high and low limits of the standard deviation.

In SS gait, the values for mean muscle activity are provided in Table 4. For SS gait, EMG data from all 21 participants were analyzed. These values were collected from EMG signals from left and right leg muscles (Figure 8 and Figure 9). The significant differences in the dual- and single-task conditions are given in the stance phase:

In this phase, each muscle had significant differences between task conditions; however, the gastrocnemius did not show any significant difference. The R BF had *p* = 0.003, d = 0.16, and L BF had *p* < 0.001, d = 0.1. The gastrocnemius exhibited no significantly different values for either side anatomically. The R TA had a small group effect (*p* < 0.001, d = 0.11) and L TA had a large group effect (*p* < 0.001, d = 0.91). The R VL had a small group effect (*p* < 0.001, d = 0.11) and L VL had a small group effect (*p* = 0.001, d = 0.13).

## 4. Discussion

The purpose of this study was to investigate the muscle activity changes involved under two different task conditions, DT and ST, while performing SS gait and GI. We recruited 21 healthy young adults and conducted research to measure muscle activity in the TA, BF, VL, and gastrocnemius using wearable EMG sensors. The hypothesis states that, in healthy young adults, there will be reduced muscle activity in the lower extremities, both during SS gait and GI when performing dual-task walking compared with ST walking. The study outcomes confirm that there were muscle activity changes caused by the DT effects. Regarding muscle activation in lower muscles during SS gait, there were statistically significant decreases for all muscles, except for the gastrocnemius, in the dual-task condition. Regarding muscle activation during GI on lower muscles, there were differences between trial groups during each phase of GI. The overall GI phase showed decreases under the dual-task state. The coactivation for lower muscles in the GI phase was shown to increase for stance BF/VL and swing G/TA. These altered muscle patterns can be considered a potential indicator of a decline in aging adult cognitive aptitude to smoothly perform motor functions.

GI muscle activation was assessed by phases from onset, weight transition, offset, and full GI. For onset muscle activation, stance BF had a medium difference between the two groups (d = 0.50). In weight transition muscle activation, stance G had a medium difference between the two groups (d = 0.49). In offset muscle activation, stance BF (*p* = 0.038, d = 0.59) and stance TA (*p* < 0.001, d = 1.1) had significant changes. In addition, in the offset phase, stance G had medium difference between two groups (d = 0.52). The full GI shows significant changes in stance TA (*p* = 0.003, d = 0.91).

GI coactivation was quantified in phases from onset, weight transition, offset, and full GI. For onset muscle coactivation, there were no significant changes. In weight transition muscle coactivation, stance BF/VL (*p* = 0.004, d = 0.88) and stance gastrocnemius/TA (*p* = 0.043, d = 0.60) had significant changes. In addition, swing BF/VL had a large difference between the two groups (group effect = 0.73). In offset muscle coactivation, stance gastrocnemius/TA had *p* < 0.001 (group effect = 1.1). The full GI shows that stance gastrocnemius/TA had *p* = 0.006 (group effect = 0.84).

SS gait muscle activation was reduced in the lower muscles in the stance phase of the gait cycle. The BF (left, *p* = 0.003; right, *p* < 0.001), TA (left, *p* < 0.001; right, *p* < 0.001), and VL (left, *p* = 0.001; right, *p* < 0.001) were significantly different between the ST and ST. In the L TA, there was a large difference between the two groups (d = 0.91). The gastrocnemius muscle had no significant change on either side of the limb.

### 4.1. Gait Initiation Phases

The GI phases were divided into three sections from the full GI gait cycle, which provided a closer observation of the changes in muscle activation. We have found significant changes in the offset phase of GI compared with onset and weight transition [42]. The TA is known to generate forces associated with ground push-off or toe-off during this offset phase [42]. Our significant findings were consistent with stance TA muscle activity for normal older adults performing GI [42]. However, we did not find significance in the gastrocnemius muscle activity, which was reported in [42]. The stance limb for each muscle showed higher muscle activation than the swing limb, setting a similar response seen in older adults [42], which may be understood as a normal healthy type of body response in GI. Alternatively, the stance BF muscle activation became significant in the offset phase, which could indicate a center of mass load response from the prior weight shift occurring from the weight transition phase [43]. A study found that young and older adults use the same motor programming to perform GI [44]. Previous studies show there are somewhat similar muscle firing patterns in old and young adults, though older adults tend to inhibit their gastrocnemius muscle activation. Thus, older adults possibly rely on TA activation when controlling the forward acceleration of the posture center of pressure [39,44].

During the DT, the mean muscle activation was reduced in all phases, including the full GI. For the onset phase, muscle activation in both stance and swing decreased, except for swing VL. For weight transition, there was less activation in swing BF, stance gastrocnemius, swing TA, and stance TA. For the offset phase, the swing BF, stance BF, swing gastrocnemius, stance gastrocnemius, swing TA, stance TA, and stance VL showed decreased activation. Khanmohammadi et al. reported that diminished levels of muscle activity in the weight transitioning phase were caused by a center of pressure shift performed in older adults switching their stance limb to the swing limb [39]. The muscles associated with the shift are VL and TA. Currently, there is a gap in literature sources discussing both GI and DT muscle activation.

In coactivation, the coordinating muscle contractions between BF and VL or TA and gastrocnemius were investigated, which share an agonist and antagonist muscle relationship. There were several GI phases that provided significant findings for muscle coactivation. The weight transition and offset phases contributed to the significance found during the full GI phase. The weight transition and offset phases showed significance on the stance limb of the gastrocnemius and TA contractions, which was also seen as significant in a study characterizing muscle activity patterns in older and younger adults’ coactivation [39]. In that study, they reported locomotor, which we referred to as the “offset phase”. Here, the study linked increased coactivity as being involved with a compensation strategy that the older adults employed to maintain posture that was in response to reduced muscular power from involuntary contractions [39].

Considering DT, the muscle coactivity across GI phases increased. For the onset phase, swing BF/VL increased for coactivation. For weight transition, swing BF/VL increased. For the offset phase, swing gastrocnemius/TA increased. Saraiva et al. reported that young adults reallocated their cognitive resources while performing a standing task [45]. This could imply that DT presents a cognitive load that interferes with the motor function response. Currently, there is a need for further study in coactivation and DT during GI to provide clearer context to the reasoning behind the associated mechanisms.

### 4.2. Steady-State Gait

In the stance phase of SS gait, all muscles involved during the DT exhibited lower mean muscle activation (Table 4). A previous study comparing young and older adults connected these populations by the gap in cognitive response to execute motor movement [46]. Dual-tasking diverts signals that are involved with performing motor function in the somatosensory system to active thought, which is deemed to be demanding, causing spatial interference [46,47]. In healthy young adults, BF, TA, and VL have been primary forward-propelling muscles used in step-by-step cyclic walking.

### 4.3. Potential Benefits and Implications

This study holds the potential to yield several significant benefits and implications. First and foremost, it can contribute to a deeper comprehension of gait control, particularly during gait initiation, by shedding light on the complex interplay between cognitive processes and motor tasks. This knowledge, in turn, can have practical applications in clinical settings, benefiting individuals with neurological disorders, musculoskeletal conditions, or those at risk of falls. Furthermore, as the population continues to age, these findings can be vital for helping older adults to maintain their mobility and independence. Beyond healthcare, the study’s insights may inform DT training programs, impact safety and ergonomics in various industries, and influence the development of wearable technology for gait monitoring. Additionally, this research has the potential to bridge disciplines, providing cross-disciplinary insights into neuroscience, biomechanics, psychology, and rehabilitation. It can also serve as benchmark data for healthy young adults, facilitating comparisons across different populations and guiding future studies.

### 4.4. Limitation

It is important to acknowledge the limitations of this study. Firstly, we measured muscle activities from the skin surface, which may introduce some discrepancies compared with direct muscle measurements. While surface EMG is commonly used in sensors and health science communities and a practical method for assessing muscle activity [48,49,50,51], there may be a potential for variations in results due to EMG sensor placement. Secondly, a limitation of our study is the absence of data regarding the difficulty level of the DT condition and whether participants had previous experience with DT exercises. These factors could potentially influence gait patterns and should be considered in future research. These limitations underline the need for further studies to address these factors and enhance the understanding of their impact on gait patterns.

## 5. Conclusions

In conclusion, our findings suggest that DT leads to decreased muscle activity in both SS gait and GI. Muscles such as BF, VL, and TA are required to generate smooth gait movements in SS gait. On the other hand, the TA, gastrocnemius, and BF are required for stable posture during GI. Overall, there is a consensus in the field that additional research is essential to narrow the potential causes of falls in older adults. Some ideas considered were rehabilitation balance training exercises, investigating joint kinematics, and further examining neuromotor interference [39,44,52].

## Figures and Tables

**Table 1:** Subject demographics.

**Measurements**	mean ± standard deviation
**Subjects (n)**	21
**Males (n)**	14
**Age (years)**	21.9 ± 3.3
**Height (m)**	1.69 ± 0.06
**Weight (kg)**	71.26 ± 9.01
**BMI (kg/m²)**	24.4 ± 2.38
**MOCA (out of 30)**	28.09 ± 1.54

**Table 2:** Mean muscle activation (%) during GI.

Phase	Muscle	ST GI	DT GI	*p*-Value	Group Effect (d)
Onset	Swing BF	5.87 ± 4.05	5.13 ± 2.23	0.795	0.07
	Stance BF	5.66 ± 3.93	4.33 ± 2.53	0.071	0.50
	Swing gastrocnemius	7.05 ± 5.84	6.56 ± 4.26	0.983	0.01
	Stance gastrocnemius	11.80 ± 14.94	8.67 ± 8.63	0.345	0.26
	Swing TA	6.31 ± 6.60	5.23 ± 4.04	0.908	0.03
	Stance TA	5.03 ± 4.06	4.26 ± 2.41	0.566	0.15
	Swing VL	5.49 ± 4.38	6.98 ± 5.93	0.266	0.30
	Stance VL	7.27 ± 7.67	6.59 ± 4.62	0.877	0.04
Weight Transition	Swing BF	6.04 ± 3.89	5.35 ± 2.76	0.362	0.24
	Stance BF	5.83 ± 3.77	6.15 ± 3.54	0.364	0.24
	Swing gastrocnemius	7.92 ± 6.70	8.11 ± 6.76	0.813	0.06
	Stance gastrocnemius	20.98 ± 33.15	11.81 ± 11.48	0.088	0.49
	Swing TA	7.46 ± 5.83	6.99 ± 5.37	0.854	0.05
	Stance TA	13.17 ± 16.10	8.25 ± 5.00	0.173	0.37
	Swing VL	4.84 ± 3.05	6.86 ± 5.46	0.123	0.42
	Stance VL	10.98 ± 8.83	11.04 ± 8.40	0.874	0.04
Offset	Swing BF	7.89 ± 5.10	6.70 ± 3.88	0.220	0.33
	Stance BF	11.61 ± 13.32	10.32 ± 14.06	0.038 *	0.59
	Swing gastrocnemius	12.51 ± 15.40	12.48 ± 15.44	0.885	0.04
	Stance gastrocnemius	47.55 ± 40.89	36.92 ± 29.90	0.074	0.52
	Swing TA	14.27 ± 6.93	14.12 ± 6.55	0.957	0.01
	Stance TA	23.94 ± 19.65	17.33 ± 17.33	<0.001 *	1.1
	Swing VL	7.86 ± 5.00	8.22 ± 5.76	0.792	0.07
	Stance VL	8.84 ± 6.81	8.35 ± 6.10	0.584	0.15
Full GI	Swing BF	6.87 ± 3.90	6.01 ± 3.00	0.201	0.35
	Stance BF	8.35 ± 6.35	7.77 ± 7.41	0.162	0.38
	Swing gastrocnemius	9.88 ± 9.25	7.28 ± 4.89	0.445	0.20
	Stance gastrocnemius	36.40 ± 46.47	27.21 ± 25.03	0.216	0.35
	Swing TA	10.48 ± 5.41	9.85 ± 4.16	0.809	0.06
	Stance TA	16.45 ± 13.47	12.73 ± 11.02	0.003 *	0.91
	Swing VL	4.20 ± 1.30	5.36 ± 2.87	0.101	0.45
	Stance VL	6.34 ± 4.01	5.58 ± 3.49	0.163	0.38

Note: d = Small (<0.2), Medium (>0.5), Large (>0.8); * *p*-value < 0.05.

**Table 3:** Mean muscle coactivation (%) during GI.

Phase	Muscle	ST GI	DT	*p*-Value	Group Effect (d)
Onset	Swing BF/VL	0.33 ± 0.08	0.36 ± 0.14	0.852	0.22
	Stance BF/VL	0.35 ± 0.10	0.34 ± 0.09	0.852	0.043
	Swing gastrocnemius/TA	0.44 ± 0.14	0.42 ± 0.11	0.625	0.13
	Stance gastrocnemius/TA	0.44 ± 0.16	0.38 ± 0.11	0.093	0.47
Weight Transition	Swing BF/VL	0.34 ± 0.10	0.47 ± 0.15	0.140	0.73
	Stance BF/VL	0.45 ± 0.18	0.33 ± 0.09	0.004 *	0.88
	Swing gastrocnemius/TA	0.45 ± 0.14	0.45 ± 0.11	0.878	0.042
	Stance gastrocnemius/TA	0.62 ± 0.22	0.48 ± 0.14	0.043 *	0.60
Offset	Swing BF/VL	0.48 ± 0.15	0.44 ± 0.12	0.220	0.33
	Stance BF/VL	0.46 ± 0.19	0.46 ± 0.18	0.852	0.049
	Swing gastrocnemius/TA	0.50 ± 0.15	0.59 ± 0.31	0.512	0.17
	Stance gastrocnemius/TA	1.78 ± 0.46	1.25 ± 0.47	<0.001 *	1.1
Full GI	Swing BF/VL	0.41 ± 0.10	0.39 ± 0.10	0.645	0.12
	Stance BF/VL	0.43 ± 0.14	0.44 ± 0.14	0.999	0.00
	Swing gastrocnemius/TA	0.47 ± 0.11	0.48 ± 0.15	0.963	0.01
	Stance gastrocnemius/TA	1.11 ± 0.27	0.90 ± 0.28	0.006 *	0.84

Note: d = Small (<0.2), Medium (>0.5), Large (>0.8); * *p*-value < 0.05.

**Table 4:** Mean muscle activation (%) during SS gait.

Muscle	ST Gait	DT Gait	*p*-Value	Group Effect (d)
Right BF	17.21 ± 11.64	12.43 ± 7.79	0.003 *	0.16
Left BF	13.94 ± 11.72	10.71 ± 9.83	<0.001 *	0.1
Right gastrocnemius	31.77 ± 17.99	28.91 ± 17.77	0.7	0.1
Left gastrocnemius	49.2 ± 58.79	45.31 ± 47.39	0.45	0.21
Right TA	44.43 ± 69.01	34.26 ± 50.81	<0.001 *	0.11
Left TA	30.06 ± 23.52	24.94 ± 18.72	<0.001 *	0.91
Right VL	15.34 ± 12.92	11.23 ± 9.66	<0.001 *	0.11
Left VL	12.87 ± 9.07	9.93 ± 6.59	0.001 *	0.13

Note: d = Small (<0.2), Medium (>0.5), Large (> 0.8); * *p*-value < 0.05.

## Data Availability

The presented data are available upon request and the approval of the corresponding author.
